# REX-001, a BM-MNC Enriched Solution, Induces Revascularization of Ischemic Tissues in a Murine Model of Chronic Limb-Threatening Ischemia

**DOI:** 10.3389/fcell.2020.602837

**Published:** 2020-12-09

**Authors:** Marta Rojas-Torres, Margarita Jiménez-Palomares, Javier Martín-Ramírez, Lucía Beltrán-Camacho, Ismael Sánchez-Gomar, Sara Eslava-Alcon, Antonio Rosal-Vela, Sandra Gavaldá, Mª Carmen Durán-Ruiz

**Affiliations:** ^1^Biomedicine, Biotechnology and Public Health Department, Cádiz University, Cádiz, Spain; ^2^Institute of Research and Innovation in Biomedical Sciences of Cadiz (INIBICA), Cádiz, Spain; ^3^R&D Department at Rexgenero Biosciences Sociedad Limitada (SL), Seville, Spain

**Keywords:** Chronic limb-threatening ischemia, critical limb ischemia, stem cell therapy, revascularization, angiogenesis, BM-MNC, bio-distribution assay

## Abstract

**Background:** Bone Marrow Mononuclear Cells (BM-MNC) constitute a promising alternative for the treatment of Chronic Limb-Threatening ischemia (CLTI), a disease characterized by extensive blockade of peripheral arteries, clinically presenting as excruciating pain at rest and ischemic ulcers which may lead to gangrene and amputation. BM-MNC implantation has shown to be efficient in promoting angiogenesis and ameliorating ischemic symptoms in CLTI patients. However, the variability seen between clinical trials makes necessary a further understanding of the mechanisms of action of BM-MNC, and moreover, to improve trial characteristics such as endpoints, inclusion/exclusion criteria or drug product compositions, in order to implement their use as stem-cell therapy.

**Materials:** Herein, the effect of REX-001, a human-BM derived cell suspension enriched for mononuclear cells, granulocytes and CD34+ cells, has been assessed in a murine model of CLTI. In addition, a REX-001 placebo solution containing BM-derived red blood cells (BM-RBCs) was also tested. Thus, 24 h after double ligation of the femoral artery, REX-001 and placebo were administrated intramuscularly to Balb-c nude mice (n:51) and follow-up of ischemic symptoms (blood flow perfusion, motility, ulceration and necrosis) was carried out for 21 days. The number of vessels and vascular diameter sizes were measured within the ischemic tissues to evaluate neovascularization and arteriogenesis. Finally, several cell-tracking assays were performed to evaluate potential biodistribution of these cells.

**Results:** REX-001 induced a significant recovery of blood flow by increasing vascular density within the ischemic limbs, with no cell translocation to other organs. Moreover, cell tracking assays confirmed a decrease in the number of infused cells after 2 weeks post-injection despite on-going revascularization, suggesting a paracrine mechanism of action.

**Conclusion:** Overall, our data supported the role of REX-001 product to improve revascularization and ischemic reperfusion in CLTI.

## Introduction

Chronic limb-threatening ischemia (CLTI) results from the narrowing and obstruction of major arteries of the limb, usually correlated with the formation of atherosclerotic plaques (Conte and Vale, 2018; Uccioli et al., 2018). The incidence of CLTI is ~500–1,000 per million per year (Nehler et al., 2014), 10–15% of which are older adults (Fowkes et al., 2013). CLTI patients suffer from chronic rest pain, ischemic ulcers which may lead to gangrene, and an eventual amputation of toes or extremities. Also, due to associated comorbidities, CLTI patients are at a greater risk of experiencing myocardial and cerebral vascular infarctions (Norgren et al., 2007; Walter et al., 2011; Simpson et al., 2014; Conte and Pomposelli, 2015). Therefore, CLTI is a debilitating disease which significantly impacts patient's quality of life by leading to dependency on caregivers, permanent local wound treatment, and the chronic use of pain-relieving medications (Lawall et al., 2012).

To date, the ultimate treatment of CLTI is a surgical revascularization through bypass grafting or angioplasty, and amputations in case of non-salvageable limbs (Lichtenberg et al., 2018), although success rate of treating CLTI is highly variable and, in many situations, suboptimal (Patel, 2016). Moreover, only 30% of patients are suitable for a surgical revascularization due to high comorbidities (Adam et al., 2005; Goodney et al., 2012). As a result, amputation rates are unacceptably high in CLTI patients, typically exceeding 15–20% at 1 year of intervention and can vary according to additional comorbidities (Duff et al., 2019) such as diabetes mellitus (DM), which elevates the amputation rate to 50% (Spreen et al., 2016). In general, DM patients are at a higher risk of developing CLTI or progressing to the severest stages of the disease, due to impaired vasculogenesis and vessel remodeling mechanisms (Howangyin and Silvestre, 2014; Thiruvoipati et al., 2015).

Thus, without effective treatments for CLTI, the prevalence of this debilitating disease may remain constant or increase with time (Duff et al., 2019). It is therefore imperative to identify alternative therapies to treat CLTI, to improve the quality of life of patients by reducing the need for multiple surgeries and/or amputations.

Novel cell therapies based on the administration of bone marrow-derived mononuclear cells (BM-MNC) have become a promising alternative to conventional surgery or angioplasty for the treatment of CLTI (Huang et al., 2005; Bartsch et al., 2007; Cobellis et al., 2008; Lu et al., 2011; Davies, 2012; Fowkes et al., 2017; Kondo et al., 2018). BM-MNC consist of a heterogeneous mix of mesenchymal stem cells (MSC), hematopoietic progenitor cells (HPC), endothelial progenitor cells (EPC), immature monocytes and lymphocytes, and pluripotent stem cells (Ratajczak et al., 2008; Franz et al., 2009). Since the first implantation of autologous BM-MNC in 2002 (Tateishi-Yuyama et al., 2002), different pre-clinical and clinical studies have reported the beneficial effects of different combinations of BM-MNC in CLTI (Kalka et al., 2000; Hamano et al., 2001; Franz et al., 2009; Fujita and Kawamoto, 2017; Rigato et al., 2017). Overall, sufficient evidence has demonstrated autologous BM-MNC (aBM-MNC) therapies to be safe and effective in promoting new vessel formation, and thus, reversal of CLTI (Fadini et al., 2010; Idei et al., 2011; Murphy et al., 2011; Liang et al., 2016; Guo et al., 2018; Wahid et al., 2018), through improved perfusion, ankle brachial index (ABI), wound healing, pain at rest, pain free walking distance, and amputation free survival (Amann et al., 2009; Cobellis et al., 2010; Fadini et al., 2010; Ruiz-Salmeron et al., 2011; Yusoff et al., 2019). However, the results found in clinical trials are variable and moreover, cell survival is usually poor under ischemic environments (Brenes et al., 2012; MacAskill et al., 2018; Qadura et al., 2018; Beltran-Camacho et al., 2020), being necessary a deeper understanding of the mechanism of action of BM-MNC in order to improve their use as cell therapy to reverse CLTI.

In this study we investigated the regenerative effect of REX-001, an adult human bone marrow (BM)-derived cell suspension enriched for MNC, when injected in a murine model of CLTI, in order to understand the mechanisms potentially involved in BM-MNC induced revascularization within the ischemic tissues, as well as to evaluate REX-001 potential bio-distribution after intramuscular administration.

## Materials and Methods

### Cell Isolation and Culture

REX-001 consists in a cell suspension of adult human BM derived cells enriched for MNC, containing a population of lymphocytes (20–51%), monocytes (4–22.3%) as well as granulocytes (20–67.7%) and hematopoietic stem cells expressing CD34 (1.4–10%). The final formulation of REX-001 can be found in the patent (US 2018/0055884 A1). For this study, BM-MNC were isolated from heparinized BM of healthy human donors (purchased from Hemacare, Charles-River). Briefly, manufacturing is performed with an initial BM volume reduction, including plasma, and red blood cell (RBC) removal. The intermediate sample bag containing volume-reduced BM goes through an automated density gradient centrifugation, followed by two washes of the MNC suspension. Approximately about 45 ml of BM-MNC product is collected in the output bag and the other components are removed to the disposable bag. The drug substance is centrifuged and the pellet is resuspended in the final formulation mix (hereafter adjuvant), a lactated ringer's solution with 1% w/v HSA and 2.5% w/v glucose in a volume of 5–30 ml.

On the other hand, the REX-001 Placebo Product (hereafter Placebo), a cell suspension of BM-derived red blood cells (BM-RBCs), is also collected from the RBC fraction during volume reduction step, formulated in 20 ml of adjuvant solution. Placebo is visually indistinguishable from fresh active product.

### Animals

**Figure 1:**
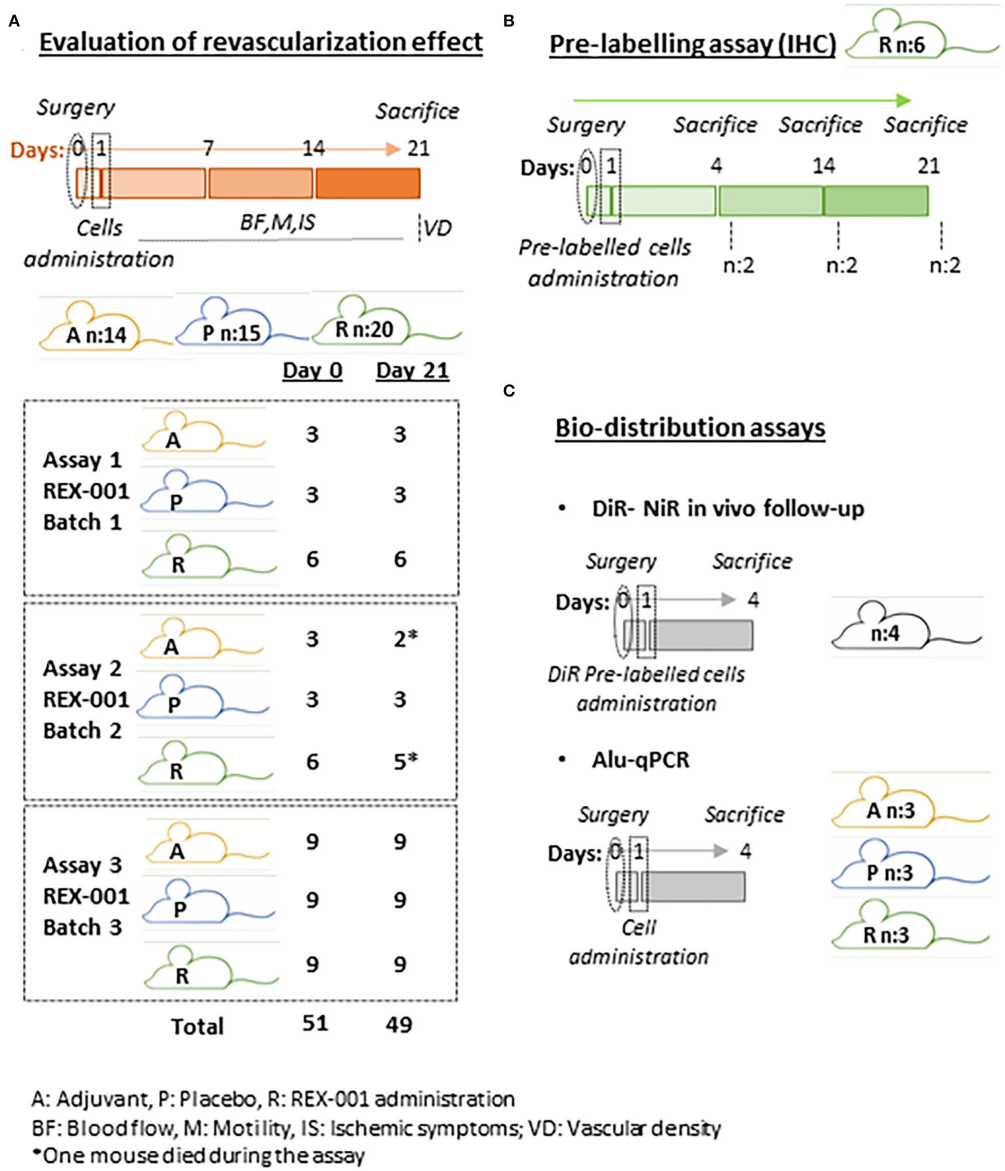
Schematic representation of experimental distribution. **(A)** Schematic representation of evaluation of revascularization effect assay distribution. Three independent assays were performed equally with REX-001 cells obtained from healthy human batches. Balb-c nude received either adjuvant solution **(A)**, placebo (P) or REX-001 cells (R). **(B)** A pre-labeling assay was performed to confirm the presence of REX-001 cells within the ischemic tissues, sacrificing Balb-c mice at different times, as shown. **(C)** Finally, REX-001 biodistribution was evaluated after intramuscular administration with DiR cell pre-labeling and NiR *in vivo* and *ex vivo* detection, and also by detection of specific human Alu sequences by qPCR in several organs (limbs, spleen, kidneys, lungs, liver). The number of mice per assay and total numbers per group is shown.

Female Balb-C Nude (CAnN.Cg-*Foxn1*^*nu*^/Crl) mice (n:70) (see Figure 1 for mice distribution), age 9 weeks, were obtained from Charles River Laboratories. Mice were allocated in individual ventilation cages inside special monitored rooms. Animals were fed sterile standard chow diet ad libitum and had free access to sterile water. Additionally, animals were constantly monitored for signs of ill-health for euthanasia in case of excessive suffering or presence of symptoms which would likely affect the experiment results. No animal was sacrificed prematurely during the experiment. Animal experiments were approved by the Ethical committee of the University of Cadiz, as well as the Andalusian Committee of animal experimentation (registration number ES110120000210 and project number 07-04-2016-043). This study followed the standard guidelines for animal research included in the Spanish laws included into the RD 53/2013 as well as the European Regulations (2012/707/UE).

### Experimental Design

In order to evaluate the effect of REX-001 on revascularization in a murine model of CLTI, and also determine the presence of these cells within the ischemic tissues and/or their bio-distribution, different assays were carried out, as described in Figure 1.

### Evaluation of Revascularization Effect

Briefly, three separated assays using REX-001 cell suspensions from healthy human donors were performed, with the same strategy and follow-up in all of them (Figure 1A). For the CLTI model, Balb-c nude mice (n:51) were anesthetized with ketamine (100 mg/kg) and xylazine (10 mg/kg) administered intraperitoneally before surgery, and double ligation of the left femoral artery was performed, occluding the distal and proximal ends with double knots (non-absorbable 6/0) of suture, as described (Niiyama et al., 2009; Beltran-Camacho et al., 2020). Mice received Ketoprofen (2 mg/kg) intraperitoneally as analgesic for three consecutive days.

Mice were then equally distributed between groups, based on blood flow levels and ischemic status registered 24 h after surgery, prior cell administration. Thus, mice received either REX-001 cells (an equivalent amount of REX-001 to obtain 1·10^6^ MNC) in 50 μl of adjuvant solution (R n:21), or 50 μl of the Placebo solution (P, n:15). In addition, another set of mice received 50 μl of the adjuvant, vehicle solution (A, n:15). Administration was done through 3-4 intramuscular injections in different sites of the left limb muscles: low back, low frontal, and middle muscles. Two mice died a few days later after infusion (from the A and R groups), and no measurements were registered from them. The total number and distribution of mice within the groups/assay can be found in Figure 1A.

### Follow up of Physiological Changes in Response to Hind Limb Ischemia and Cell Administration

Blood flow was measured for both paws, on day 0 (before and after surgery), day 1, day 7, day 14, and day 21, using a Laser Doppler system (Periflux System 5000; Perimed). The right limb was taken as control, not-injured limb, and perfusion was expressed as the ratio of left (ischemic) vs. right (non-ischemic) limb. In addition, ischemic symptoms such as motility impairment, inflammation, ulceration, and necrosis were also registered for all mice during the entire assay according to Tarlov and ischemia scores (Tarlov, 1954; Yu et al., 2005; Brenes et al., 2012), registered in Supplementary Tables S1–S3.

### Tissue Extraction and Processing

Mice (n:49) were sacrificed in a CO_2_ chamber on day 21 after surgery. Low frontal muscles (tibialis) and middle muscles (bicep femoris, adductor, and semi-membranous) of the left limb were extracted and fixed for 15 days embedded in 4% formaldehyde prior dehydration in 30% sucrose during 24 h. Tissues were then frozen in OCT before immunohistochemistry (IHC).

### Cell Pre-Labeling Assay

In addition to the groups described in section Evaluation of revascularization effect, another set of Balb-C Nude mice (n:6) were employed to confirm the presence of human cells within the injured area, by using a pre-labeling approach (Figure 1B). Thus, REX-001 cells were pre-labeled with biocompatible organic fluorescent nanoparticles (LuminiCell Tracker™ 540, SCT010 Sigma-Aldrich), 1 h at 37°C, 5% CO_2_, and washed several times with PBS 1X before being administered (1·10^6^ cells/mouse) to mice that underwent femoral ligation 24 h earlier, as described above. Mice were sacrificed in a CO_2_ chamber at different times after surgery: day 4 (n:2), day 14 (n:2), and day 21 (n:2), and muscles from low frontal limb and medium limb were extracted and processed as described before.

### Immunohistochemistry (IHC)

Different tissue sections from all mice were employed to calculate the number of vessels and diameter size changes in response to cell administration and also to detect pre-labeled human cells within the ischemic limbs. Thus, tissues embedded in OCT were cut in consecutive sections of 8 μm and placed in poly-lysine slides. In total, 5 tissue sections (low frontal), separated by 32 μm each, were employed for cellular and vessel detection, while 3 tissue sections (middle muscles) were used to measure vascular diameters. All sections were pretreated for antigen retrieval and permeabilization with 1% SDS during 5 min and with 1% triton, 20 min, followed by a blocking step with 5% goat serum (S-1000, Vector Laboratories) and 0.1% triton for 1 h. Tissues were then incubated with anti-α-actin smooth muscle (α-SMA, 1:400, A5228 Sigma-Aldrich) at 4°C, over-night. Tissue auto-fluorescence was prevented by incubation with 0.3% Sudan Black B-70% ethanol for 20 min and incubation with specific secondary antibodies for 1 h in the darkness at room temperature (RT) were performed. Finally, nuclear staining with DAPI (0.2 μg/ml) was carried out.

The entire tissue area of each section was analyzed by fluorescence microscopy, acquiring images at 20x and 40x using MMI CellCut Plus (Olympus) and visualizing them with the Zen 2 (Zeiss) software. Results were expressed as the number of blood vessels per cm^2^ or blood vessels diameter (μm). Additionally, the number of vessels containing pre-labeled cells vs. the total number of vessels were also quantified, as described (van Weel et al., 2008; Beltran-Camacho et al., 2020). Results were expressed as the mean ± SEM.

### Bio-Distribution Assay

In order to confirm the presence of human (h) cells within the ischemic tissues and moreover, evaluate potential bio-distribution after intramuscular administration, two complementary approaches were carried out (Figure 1C): the application of an *in vivo* assay labeling cells with the lipophilic dye 1,1-dioctadecyl 3,3,3,3 tetra-methyl-indo-tricarbocyanine iodide (DiR) and Near Infrared (NiR) detection (Kalchenko et al., 2006; Bulte and Daldrup-Link, 2018), and further quantification of human DNA (hu-DNA) by q-PCR with specific Alu sequences (Funakoshi et al., 2017).

#### DiR Labeling

REX-001 cells (3·10^6^) were labeled with 6.67 μM of DiR (Biotium #60017) according to manufacturer's instructions, 25 min at 37°C, 5% CO_2_, centrifuged and washed three times with PBS 1X to discard excess or unbound dye. The final cell pellet was then resuspended in 50 μl adjuvant solution before being administered intramuscularly to Balb-c nude (n:3) mice 24 h after femoral ligation, as described before (1·10^6^/mouse). In addition, a negative control consisting in a mouse injected with unlabeled cells was used to discard any background signal. Anesthetized mice were scanned using NiR LI-COR Odyssey system (LI-COR) at days 1 and 4 after cell infusion. Parameters used for the scan: intensity 3, detectors in 700 nm and 800 nm activated, ~30 min. Mice were sacrificed on day 4 and organs (lungs, kidneys, liver, spleen, right, and left limb muscles) were extracted and scanned again *ex vivo* to evaluate the presence of cells in individual organs. Intensity values were normalized vs. the negative control, taken their background signal as cero.

#### Alu-Based qPCR Quantitative Assay

Finally, the presence of human cells in different organs was also quantified by amplification by qPCR of Alu specific sequences (Figure 1C), as described (Beltran-Camacho et al., 2020). Thus, 24 h after femoral ligation, another set of CLTI mice (n:9) received intramuscularly 50 μl of adjuvant solution (A, n:3), placebo solution (P, n:3) or 1·10^6^ REX-001 cells (R, n:3). Animals were sacrificed 4 days after cell transplantation and organs (lungs, kidneys, liver, spleen, right, and left limb muscles) were extracted, directly frozen in liquid N_2_ and stored at −80°C. Biopsies (≈ 40–50 mg) were crushed using liquid N_2_ into a fine powder. Genomic DNA was isolated from tissue samples using the E.Z.N.A.® Tissue DNA Kit (Omega-biotek D3396-01). Human DNA (hu-DNA) was quantified using the Alu detection approach, as described (Funakoshi et al., 2017; Beltran-Camacho et al., 2020).

Each qPCR reaction employed 100 ng of genomic DNA in which Alu sequences were amplified using TaqMan Universal Master Mix II (ThermoFisher 4440043), 0.2 μM primers and 0.25 μM hydrolysis probes designed by (Funakoshi et al., 2017): forward primer 5′-GGTGAAACCCCGTCTCTACT-3′, reverse primer 5′-GGTTCAAGCGATTCTCCTGC-3′ and label probe 5′-(6-FAM)-CGCCCGGCTAATTTTTGTAT-(BHQ-1)-3′ (synthesized by Metabion). qPCR was carried out using a CFX Connect Real-Time System (Biorad) with the following protocol: 1 cycle of 95°C/10 min and 50 cycles of 95°C/15 s, 56°C/30 s, and 72°C/30 s.

Linearity and resolution limits were determined by diluting known amounts of hu-DNA (from 5 ng to 1 pg) in murine DNA, as described (Funakoshi et al., 2017; Beltran-Camacho et al., 2020). qPCR was performed in triplicates and C_t_ mean values were plotted to obtain the lineal equation as well as the *R*^2^ values (Supplementary Figure S2). The sensitivity of the assay is such that one human cell among 10,000 mouse cells could be detected.

The amount of hu-DNA detected in 100 ng of total genomic DNA extracted was measured by qPCR, and further extrapolation of the total hu-DNA extracted was then calculated per mg of tissue. Finally, the amount of DNA (ng) per cells was calculated considering the relation of 5 pg of DNA per human cell (Dolezel et al., 2003). C_t_ values were analyzed with Bio-Rad CFX manager software (Biorad). Results were expressed as the mean ± SE of human cells detected per mg of tissue.

### Statistical Analysis

Statistical analysis was performed with GraphPad Prism v.8 software. Data were verified for normal distribution using Shapiro-Wilk normality test. Experiments with two different categorical independent variables (blood flow measurements within time) were analyzed with a two-way ANOVA test and Tukey's multiple comparison test for *post-hoc* analyses. Differences between three groups was tested with either One-way ANOVA test and Tukey-s multiple comparisons test for *post-hoc* analysis or Kruskal-Wallis test and Dunn's test as *post-hoc*. Finally, the Pearson Coefficient value was calculated to check for potential correlations between the variables tested. Differences were statistically significant with *p* < 0.05.

## Results

### REX-001 Promotes a Significant Blood Flow Recovery in CLTI Mice

**Figure 2:**
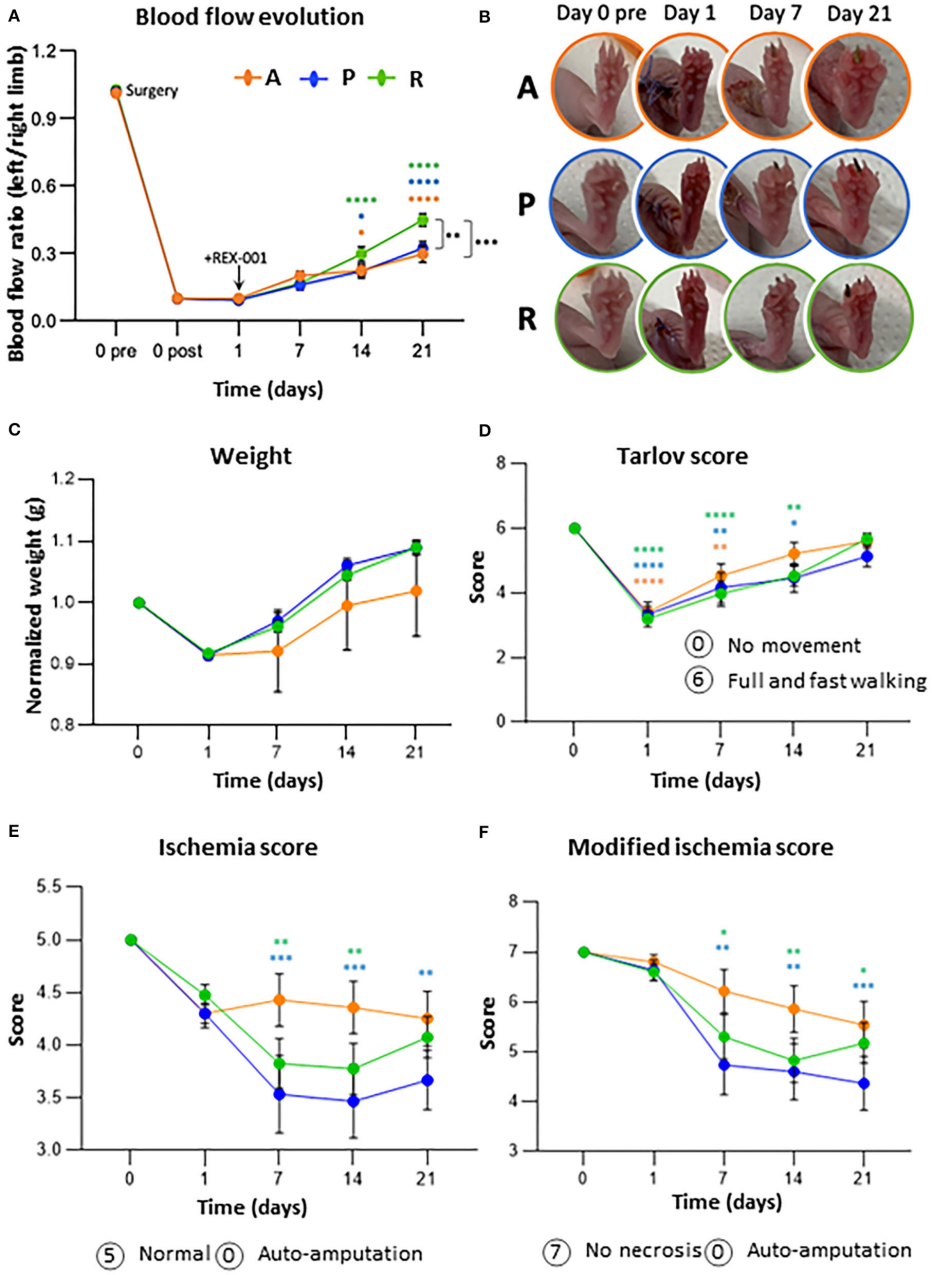
REX-001 cells enhance revascularization and blood recovery in CLTI mice. **(A)** Blood flow evolution per group within time. Perfusion (PU) averaged ratios of left (injured) vs. right (non-injured) limbs are shown. **(B)** Representative images of ischemic symptoms (inflammation, necrotic fingers) in adjuvant **(A)**, placebos (P) and REX-001 (R) treated mice. **(C)** Weight changes, normalized vs. averaged values on day 0, pre-surgery. **(D)** Motility changes within time, according to Tarlov score values. Ischemic changes (ulceration, necrosis) detected along the assay, measured with both, the Ischemia **(E)** and modified ischemia scores **(F)**. Groups tested: Balb-c nude mice injected with Adjuvant (vehicle-only) (n:14), Placebo (*P*, n:15) and REX-001 (*R*, n:20, from different donors, *R*1, n:6, *R*2, n:5, *R*3, n:9). The averaged values from the three assays is shown as mean ± SE. Complete scores meaning are described in Supplementary Tables 1–3. Significant differences were calculated by two-way ANOVA and Tukey *post-hoc*, represented as: * with colors compared to post-surgical ratios at day 0 and * in black between groups in the same day (*p-value < 0.05, **p-value < 0.01, ***p-value < 0.001, and ****p-value < 0.0001).

Immediately after femoral ligation, a significant decrease of blood flow was observed in all groups compared to pre-surgical values (>85% reduction), detecting a slight recovery in the three groups by day 7 (Figure 2A). Major changes were detected after day 14, with the group of mice treated with REX-001 (R) already showing a significantly higher recovery of blood flow (*p* < 0.0001) after surgery, compared to placebo- (P) and adjuvant-treated animals (A), whose blood flow rates also increased vs. baseline (*p* < 0.05) although remained below that of REX-001-treated mice. By day 21, differences were more pronounced, with the REX-001 group presenting blood flow ratios significantly higher than that of P group (*p*-value < 0.01) and the group of adjuvant-treated mice (*p*-value < 0.001). Remarkably, mice with placebo (P) and adjuvant solutions (A), showed similar blood flow ratios after 21 days of follow up.

In order to evaluate individual variability, coefficient of variations (CVs %) were calculated per day in each group of mice and human donor material, as indicated in Supplementary Figure S1. The highest variations were seen on day 7 (39.07–50.66% on average) and day 14 (38.10–55.06% on average). After 21 days post-surgery, mice treated with REX-001 cells showed a more defined pattern, with higher increase of blood flow ratios and lower variability (27.10%CV) than placebos and adjuvant groups (36.08 and 47.39%CV, respectively).

### Evaluation of Ischemic Symptoms

All mice were evaluated periodically, and images were taken of all mice on days 0, 1, 7, 14, and 21 (Figure 2B). In terms of body weight, no differences were observed between all groups (Figure 2C) or between different REX-001 batches, losing weight right after surgery (on day 1 they lost on average 1.5 ± 0.06 g) but with tendency to recover, and to gain weight by day 21 in the case of the REX-001 and P groups. Although the adjuvant group (A) recovered less body weight than the others, weight changes were likely due to the surgical intervention and not due to the treatment applied.

Similarly, a significant mobility impairment was observed for all mice 24 h post-surgery (*p* < 0.0001), the majority of them not bearing weight properly on the injured toe or limping in some cases, probably as a result of the inflammation related to the surgical procedure (Figure 2D). Mobility-related symptoms continued unchanged after day 7, although most mice, mainly the ones treated with REX-001 (R), appeared to recover certain grade of mobility by day 21 independent of their ischemic outcomes. Finally, in response to femoral ligation, mice began showing symptoms of inflammation and ischemia (reddish area and black nails) by day 2, progressing to black necrotic fingers in some cases (Figures 2E,F). Overall, the placebo group (P) showed the worst symptoms along the assay, while the adjuvant group (A) showed a slower progression but worsening status from day 7 onwards, with some mice losing several digits by the end of the study. REX-001 treated mice (R), however, began showing some improvement after day 7, with less inflammation and, despite showing nails or even necrotic toes, there was not worsening along the time of the assay, suggesting that cell administration might have stopped the ischemic progression.

### REX-001 Promotes Collateral Vessel Formation and Arteriogenesis

**Figure 3:**
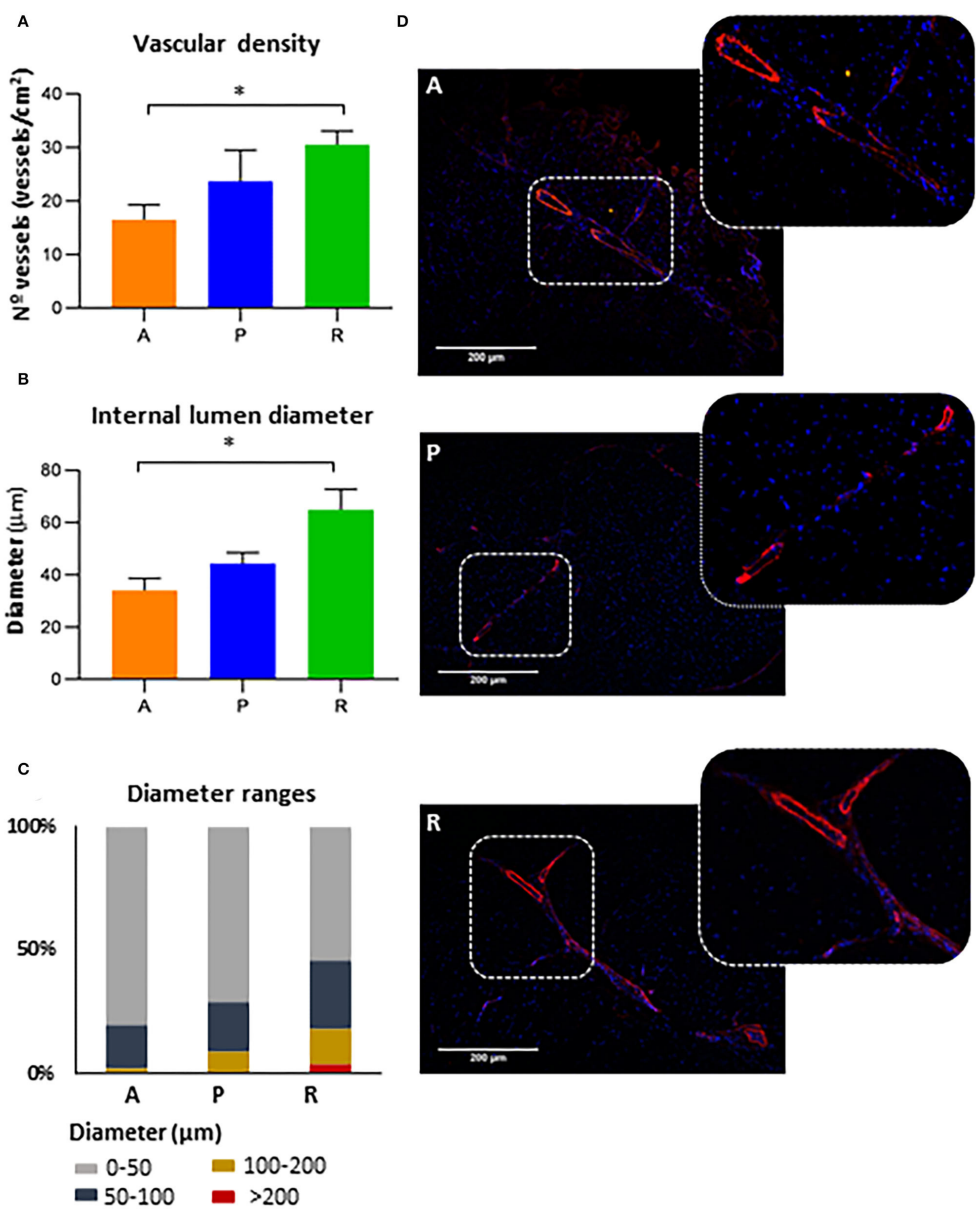
Vasculogenesis and arteriogenesis. **(A)** The number of blood vessels (vessels/cm^2^) and **(B)** diameter sizes (μm) were measured for mice administered with adjuvants (A, n:14), placebos (*P*, n:15) or REX-001 cells (*R*, n:20). **(C)** Vessel classification based on abundance percentage of different ranges of internal lumen diameter (μm). **(D)** Representative IHC images to measure vascular density and diameter size are shown, using anti-mouse smooth muscle α-actin (red) and DAPI (blue). Data were presented as mean ± SEM. Significant differences were seen between the REX-001 (*R*) and adjuvant **(A)** treated mice (*p-value < 0.05), calculated with a one-way ANOVA, and Tukey *post-hoc* tests.

We subsequently tested whether blood flow recovery was related to an increased vascularization (Figure 3). Overall, our results indicated that administration of REX-001 promoted a significant increase in the number of μ-SMA positive vessels (Figure 3A) and vessel diameter (Figure 3B) compared to the adjuvant group (*p*-value < 0.05). Conversely, the placebo group (P) showed a slight increase in the number and diameter of vessels than the adjuvant group (A), although these changes were not significant compared to adjuvants or REX-001 treated mice.

Further classification of vascular vessels per diameter size (Figure 3C), indicated that mice treated with either adjuvant or placebo solution had higher number of vessels (80.3% and 70.7% from the total diameters measured) with lower diameters (0–50 μm) compared to the REX-001 group (54.1%). Conversely, REX-001 treated mice (R) showed more vessels with larger diameters (14% ranged between 100 and 200 μm and 3.8% diameters >200 μm) than the other groups (Adjuvants, with only 1.6% of capillaries between 100 and 200 μm; Placebos, with 8.6% between 100 and 200 μm and only 0.3% of diameters >200 μm). Several examples are shown in Figure 3D.

In summary, the overall data related to vessel formation were in agreement with the results seen for blood flow after surgical ligation and ischemia, in which, by day 21, only the REX-001 group presented a significant perfusion recovery compared to the adjuvant and placebo groups (*p*-value < 0.01). Considering these data, we evaluated whether there was any relationship between vessel formation, diameter, and blood flow ratios, by applying a Pearson correlation test to the measurements obtained at day 21. Remarkably, we found significant correlations between blood-flow ratios and vessel number (*p*-value: 0.03, R: 0.599), but not between blood flow and internal diameter sizes (*p*-value: 0.21, R: 0.28) or between vascular density and diameters (*p*-value: 0.087, R: 0.372).

### REX-001 Migrates to Vasculature of Ischemic Tissues After Intramuscular Administration

**Figure 4:**
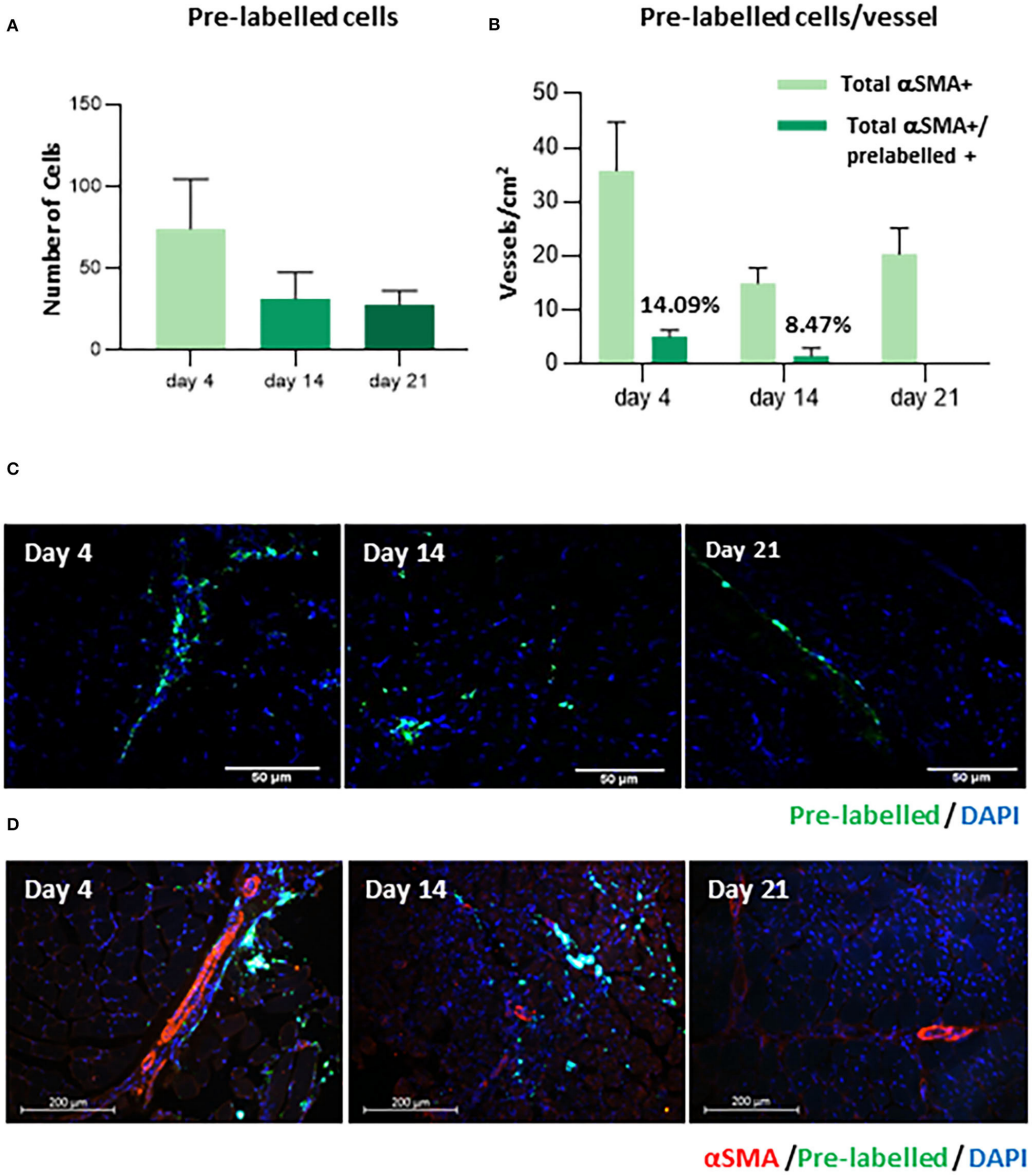
Detection of human pre-labeled BM-MNC. **(A)** The graph represents the number of cells pre-labeled with LuminiCell Tracker™ 540, detected on day 4, 14, and 21 by IHC. **(B)** The proportion of vascular vessels detected on day 4, 14, and 21, incorporating REX-001 pre-labeled cells (pre-labeled+/αSMA+) is shown. **(C)** Representative IHC images confirming the presence of pre-labeled cells (green) within the tissue and also **(D)** in the vicinity of vascular vessels (α-SMA staining, reed), are shown for mice sacrificed on day 4, 14, and day 21.

IHC assays confirmed the presence of pre-labeled REX-001 within the ischemic tissues surrounding the femoral artery, mainly on day 4, but also on day 14 and day 21 after femoral ligation, although in lower levels than at early dates (Figure 4A). Moreover, pre-labeled REX-001 were found in the vicinity of vascular vessels (pre-labeled+/αSMA+) mainly on day 4 (14.09%), with fewer numbers at day 14 (8.47%) and scarcely detected at day 21 (Figure 4B). Thus, REX-001 cells migrated to the damaged vasculature after intramuscular injection, although their numbers significantly decreased after 2–3 weeks post-injection (Figures 4C,D).

### Cell Bio-Distribution

**Figure 5:**
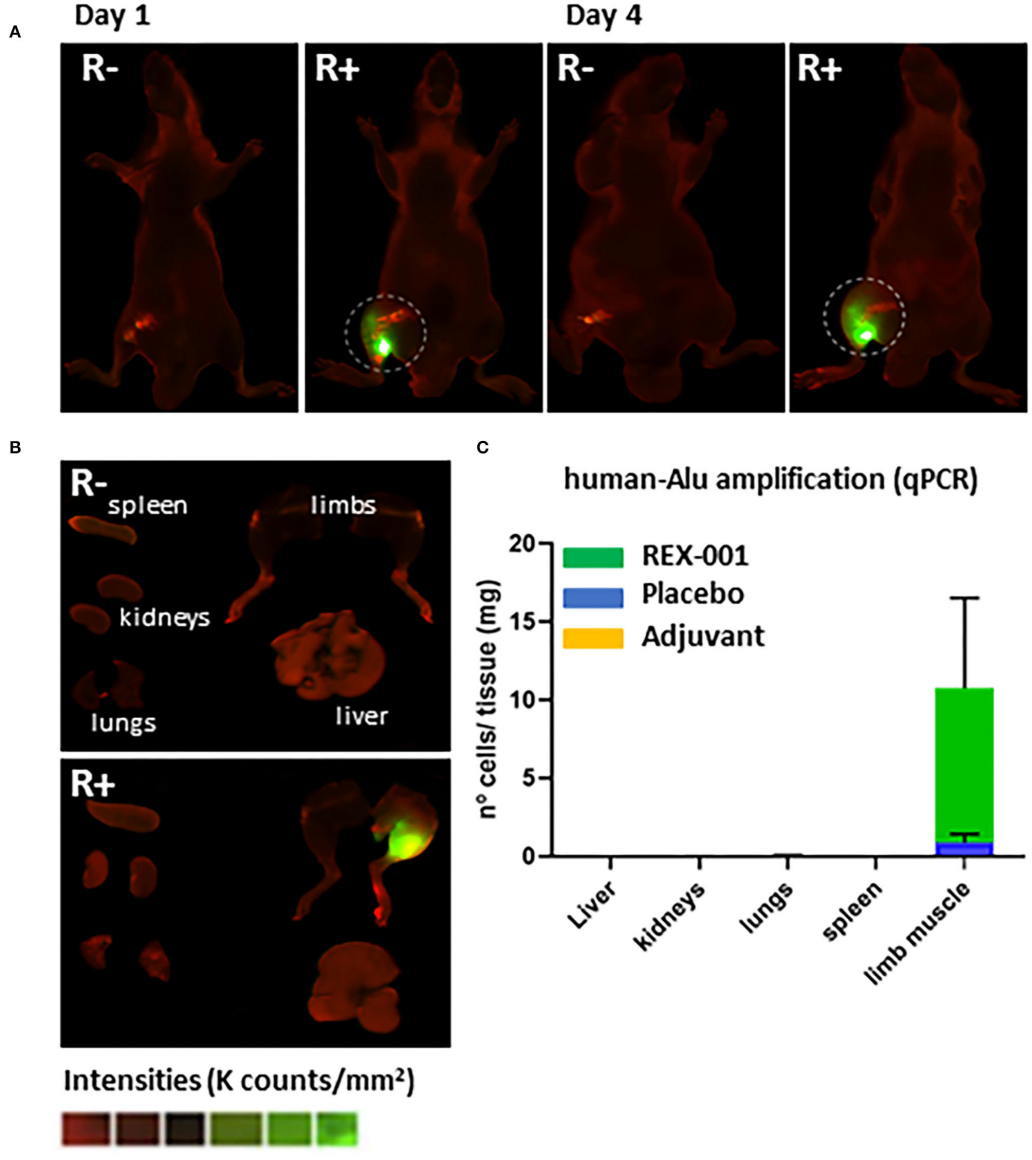
Biodistribution assay. **(A)** Representative images of NiR scanning performed *in vivo* on day 1 (after cell administration) and day 4 in Balb-c nude mice with DiR pre-labeled REX-001 (R+, n:3) or with un-labeled cells (R-), both administered intramuscularly. **(B)** Representative images of *ex vivo* NiR scanning for the organs extracted on day 4 for the R+ and R- mice (lungs, spleen, kidneys, liver, and limbs). Intensity values were calculated as K counts/mm^2^, with final R+ intensities calculated after subtracting background, negative signal from R- mice. **(C)** The number of cells detected per mg of organ tissue analyzed on day 4 (after cell administration) in Balb-c nude treated with either REX-001 cells (R, n:3), placebos (*P*, n:3) or adjuvants (A, n:3) was calculated after measuring the presence of human DNA with specific Alu sequences and qPCR analysis, in several organs (spleen, kidneys, lungs, liver, left limb).

Next, we subsequently tracked the route of migration of cells post-intramuscular administration, in order to explain the decrease in the number of cells in ischemic areas after elapsed time. Two independent cell-tracking assays were carried out. First, according to *in vivo* (Figure 5A) and *ex vivo* (Figure 5B) NiR scans, our data corroborated that after intramuscular administration, DiR-prelabelled cells were mainly allocated in the ischemic areas of the limb, with almost no presence of REX-001 4 days after cell administration in lungs, kidneys, liver nor the spleen (Figure 5B). Similarly, amplification of specific human Alu sequences by qPCR (Funakoshi et al., 2017; Beltran-Camacho et al., 2020) confirmed the presence of human DNA, mainly in the hind limb muscle (Figure 5C) (9.90 ± 5.76 cells/mg of tissue). Also, a small percentage of human DNA was detected in the hind limb when the placebo solution was administered (<1 human cells/mg tissue).

## Discussion

To date, different studies have demonstrated the potential of using autologous BM-MNC to treat CLTI patients, as a safe and efficient strategy to achieve therapeutic angiogenesis and prevent amputation (Idei et al., 2011; Murphy et al., 2011; Liang et al., 2016; Guo et al., 2018). However, the urgent need to translate BM-MNC therapy into clinic may be reflected by the few pre-clinical assays testing BM-MNC in animal models of CLTI, and moreover, by the high variability seen between clinical trials (e.g., different study designs, dosing, routes of administration). This, in turns, could explain the scarce knowledge regarding the underlying mechanisms of action of these cells as well as the cell migration routes (Fadini et al., 2010; Idei et al., 2011; Pignon et al., 2017; Qadura et al., 2018). In addition, the selection of an optimal investigational product composition (bone marrow vs. peripheral/blood origin); isolated (MSC, CD34+, and EPC) or combined (mixed *ex vivo* or selected from an original cell source, such as BM-MNC); un-stimulated or pre-conditioned with VEGF, FGF-2, G-CSF, is still under intensive research (Zhang et al., 2008; Layman et al., 2011; Brenes et al., 2012; Gremmels et al., 2014; Beegle et al., 2016; Dong et al., 2018; MacAskill et al., 2018). Therefore, major effort is required to reach a consensus regarding these factors in order to standardize cell therapy in ischemic diseases such as CLTI (Brenes et al., 2012).

REX-001, a solution enriched with human BM-derived MNC, is intended for treatment of CLTI, and is currently under investigation in a two pivotal Phase III clinical trials (NCT03174522 and NCT03111238). Herein, we have shown, for the first time, the pre-clinical results evaluating the effect of REX-001 product on revascularization in a murine model of CLTI, using the Balb-c nude strain, as well as the cellular biodistribution of this product as result of intramuscular administration. Furthermore, the comparative effect of REX-001 vs. the placebo solution, specifically designed and formulated for the double-blinded aforementioned Phase III clinical trials, has been also analyzed for the first time in an animal model.

In our study, a significant decrease of blood flow (>85% reduction) was seen after double ligation of the femoral artery, leading to inflammation and reduced mobility (most probably associated with the surgical procedure) as well as other ischemic symptoms (ulceration, necrotic digits), which were detected in most mice, with no clear differences between groups treated with either REX-001, placebos or adjuvants (vehicle only). The ischemic symptoms worsened with time, leading to progression of necrosis and digits falling off due to severe necrosis in several cases, although in general, REX-001 treated mice showed a less worsening status in the last 2 weeks, while mice in the adjuvant group showed slower disease progression, yet, presented the worst ischemic conditions on day 21. Regarding blood flow recovery, all three groups showed a slight increase of blood flow after 7 days of femoral ligation, although the REX-001 group showed a significant recovery by day 14 and 21 compared to both placebos (*p*-value < 0.01) and adjuvant treated mice (*p*-value < 0.001).

Balb-c nude mice are known to show poor perfusion recovery and slower revascularization response compared to other strains, such as C57BL/6 (Fukino et al., 2003; Nossent et al., 2017; Aref et al., 2019). As a result, the recovery seen in these mice (after 14–21 days) was slower than in other models (7–14 days), corroborating a dependency in the results with the strain used, as suggested (Fukino et al., 2003; Saqib et al., 2011; Aref et al., 2019). Nevertheless, the model applied seemed adequate to evaluate CLTI (Saqib et al., 2011; Nossent et al., 2017), and our data supports the positive effect of REX-001 product by enhancing blood flow perfusion after the ischemic injury induced.

Furthermore, the increase of blood flow in REX-001 treated mice was accompanied by a significant increase in vascular density and a higher percentage of vessels with wider diameters than placebos and moreover, than the adjuvant treated mice. The placebo solution promoted an increase of the number of vessels compared to the vehicle solution, although this was insufficient in achieving a significant recovery of blood flow. Therefore, similar to human trials, and in line with pre-clinical studies with intramuscular or intra-arterial administration of BM-MNC (Shintani et al., 2001; Yoshida et al., 2003), our data supports that administration of REX-001 promotes an increase of blood reperfusion due to increased vascular density.

We also confirmed the presence of human cells near the vascular vessels in ischemic tissues (14.9% of vessels contained REX-001 on day 4), supporting the hypothesis that cells administered intramuscularly do indeed reach the vasculature in ischemic areas adequately (Beltran-Camacho et al., 2020). Moreover, *in vivo* and *ex-vivo* DiR-pre-labeling assays and qPCR amplification of human Alu sequences, indicated that REX-001 remained in the ischemic limbs, with no apparent cell translocation to other organs. Remarkably, the fact that we detected human DNA traces of the placebo solution (a residual RBC fraction derived from the human BM initial source) in the ischemic limbs, even at very low levels, supported the sensitivity of the strategy followed.

Conversely, the percentage of REX-001 in the ischemic limbs decreased within time, with no detection 21 days post-surgery. Different studies have reported such decrease, using among others, autologous cells expressing green fluorescent protein (GFP) and firefly luciferase (Fluc) reporter genes (van der Bogt et al., 2012), with a significant cell loss from the ischemic tissues after 4 weeks (van der Bogt et al., 2012). In this sense, future efforts should be made to implement transplanted BM-MNC survival and/or the ratio of local delivery in order to enhance the revascularization orchestrated by these cells. We have demonstrated here, in agreement with several clinical trials using this approach (Higashi et al., 2004; Cobellis et al., 2008; Matoba et al., 2008; Motukuru et al., 2008; Iafrati et al., 2011), the efficiency of intramuscular administration to maximize the local concentration of stem cells in the ischemic area. Perhaps, as suggested (Davies, 2012; Qadura et al., 2018), the combination of intra-muscular and intra-arterial administration may be an even better strategy to allow stem cells to reach additional areas, at higher concentrations, including ischemic muscle regions that still are perfused (Van Tongeren et al., 2008; Franz et al., 2009) and thus, achieve a higher and faster blood perfusion recovery.

In our study, the decrease in cell numbers with time did not correlate with cell translocation to other organs, suggesting that infused REX-001 might not proliferate after having promoted angiogenesis, so these cells may not be directly involved in vessel formation (given the small percentage of REX-001+ vessels), but they most probably contribute to revascularization in a paracrine fashion (van Weel et al., 2007; Burdon et al., 2010; van der Bogt et al., 2012). In this regard, some of the cells included in REX-001 product (enriched for MNC, as well as granulocytes and hematopoietic stem cells expressing CD34) might participate in the paracrine effect. For instance, circulating BM-derived EPC (CD34+CD45+) have been described as powerful angiogenic agents (Yanishi et al., 2020). These cells release angiogenic and chemo-attractant factors once migrated into the ischemic tissues, recruiting immune cells (neutrophils, monocytes) and activating other cells that will participate in the inflammatory response and also contribute to vascular remodeling (Beltran-Camacho et al., 2020). In addition, BM-immune precursors cells are thought to have an active role in angiogenesis and/or arteriogenesis itself (Nossent et al., 2017). For instance, neutrophils not only participate in inflammation but they can also promote vascularization by inducing angiogenesis via a pro-angiogenic phenotype (Lin et al., 2017; Seignez and Phillipson, 2017; Beltran-Camacho et al., 2020).

Preliminary results obtained in our laboratory indicated that REX-001 cultured *ex vivo* releases angiogenic cytokines such as CXCL4/PF4, metalloproteinase-8 (MMP-8) or Interleukin-8 (IL-8) to the conditioned medium. Moreover, the levels of MMP-8 seem to increase exponentially after several hours of culturing these cells in basal media at 37°C, 5% CO_2_. The involvement of MMP-8 as well as other metalloproteinases in angiogenesis has been already described (Lin et al., 2008; Deryugina and Quigley, 2010; Fang et al., 2013; Quintero-Fabian et al., 2019). Studies with MMP-8 and MMP-2 knock-out mice have shown an *in vitro* diminishment of cell proliferation and neo-capillary network growth, as well as a reduction in HUVEC migration and impaired angiogenesis *in vivo* (Cheng et al., 2007; Fang et al., 2013). MMPs not only contribute to the remodeling/degradation of the extracellular matrix (ECM), but also participate in many biological processes involved in stroke, cardiovascular diseases or arthritis (Chuang et al., 2019). Moreover, macrophage activation results in MMP secretion. Remarkably, some MMPs are related to the transition from M1 to M2 macrophage phenotypes, associated with immunomodulatory processes (Berg et al., 2019), suggesting that this transition might be promoted in presence of REX-001 cells. Future assays should be performed to confirm such phenomenon. Apart from MMP-8, IL-8 was also detected as released by REX-001 *in vitro*. IL-8, also named neutrophil chemotactic factor, is a soluble chemoattractant produced mainly by macrophages, but also by other cell types such as epithelial and endothelial cells (Oude Nijhuis et al., 2003). Notably, not only does IL-8 boost phagocytosis in macrophages, it also stimulates angiogenesis (Koch et al., 1992; Wu et al., 2018; Fousek et al., 2020). Thus, although preliminary, these results indicate that REX-001 release certain factors such as MMP-8 and IL-8 that might contribute to the vascular restoration induced by this cell product. Further studies are required to confirm these data and moreover, to complete the information regarding the molecular mechanisms of action of these cells. Despite this, our results confirmed that the administration of REX-001, and therefore the combined effect of such populations, has proven to be effective in promoting revascularization after CLTI.

## Conclusion

Overall, our findings demonstrate the efficacy of REX-001 implantation to enhance blood flow recovery after ischemic injury, by inducing functional neovascularization in a murine model of CLTI. Moreover, detailed cell-tracking corroborated the efficiency of intramuscular administration, with REX-001 exerting a focalized action within the ischemic tissues and no apparent translocation to other organs. The decrease seen in cell numbers within time, despite promoting an increased revascularization, suggests a paracrine mechanism of action for these cells. Future research should now be focused on analyzing the molecular mechanisms of action of REX-001 cells as well as to evaluate their effect at the clinical level.

## Data Availability Statement

The original contributions presented in the study are included in the article/Supplementary Materials, further inquiries can be directed to the corresponding authors.

## Ethics Statement

The animal study was reviewed and approved by Animal experiments were approved by the Ethical committee of the University of Cadiz, as well as the Andalusian Committee of animal experimentation (registration number ES110120000210 and project number 07-04-2016-043).

## Author Contributions

MR-T and MJ-P: experimental assays, collection and assembly of data, data analysis and interpretation, manuscript writing, and final approval of manuscript. LB-C: support in experimental assays and data collection, manuscript writing, and data analysis and interpretation. IS-G, AR-V, and SE-A: support in animal experimental assays and data collection. JM-R and SG: provision of study materials, conception, design, data analysis and interpretation, and final approval of manuscript. MD-R: conception and design, financial support, administrative support, collection and assembly of data, data analysis and interpretation, manuscript writing, and final approval of manuscript. All authors contributed to the article and approved the submitted version.

## Conflict of Interest

MD-R, MR-T, and MJ-P collaborated with Rexgenero Biosciences SL in the project, supported by Rexgenero, where the current results were obtained from. JM-R and IS-G are employees of Rexgenero Biosciences SL. The remaining authors declare that the research was conducted in the absence of any commercial or financial relationships that could be construed as a potential conflict of interest.

## Supplementary Material

Supplementary Materials includes Supplementary Tables 1–3, with Tarlov scores (motility changes), ischemia, and modified ischemia scores, respectively; Supplementary Figure 1 (with individual variations in blood flow) and Supplementary Figure 2 (with linear equation applied to calculate human DNA).
