# Supplementary material for: REX-001, a BM-MNC Enriched Solution, Induces Revascularization of Ischemic Tissues in a Murine Model of Chronic Limb-Threatening Ischemia
Source: Front Cell Dev Biol. 2020 Dec 9;8:602837. doi: 10.3389/fcell.2020.602837 (PMC7755609; doi:10.3389/fcell.2020.602837)
Supplement: Supplementary file 1 [file Data_Sheet_1.PDF]

Supplementary Material

"REX-001, a BM-MNC enriched solution, induces revascularization of ischemic tissues in a murine model of chronic limb-threatening ischemia"- Rojas-Torres M et al.

1 Supplementary Figures and Tables

1.1 Supplementary Tables

Supplementary table 1. Tarlov score [1, 2]. Functional scoring to evaluated motility, leg movement and difficulty walking

**Tarlov Score**	
0	No movement
1	Barely perceptible movement, non-weight bearing
2	Frequent movement, non-weight bearing
3	Support weight, partial weight beaing
4	Walks with mild déficit
5	Normal but slow waking
6	Full and fast walking

Supplementary table 2. Ischemia score [1]. Functional scoring to evaluated ischemic symptoms, advance of the characteristics along the leg

**Ischemia Score**	
0	Auto-amputation > half lower limb
1	Gangrenous tissue > half foot
2	Gangrenous tissue < half foot, with lower limb muscle necrosis
3	Gangrenous tissue < hal foot, without lower limb muscle necrosis
4	Pale foot or gait abnormalities
5	Normal

Supplementary table 3. Modified ischemia score [1]. Functional scoring to evaluated ischemic symptoms, advance of the characteristics along the leg, modified to be more correctly for mice

Modified Ischemia Score	
0	Auto-amputation of leg
1	Leg necrosis
2	Foot necrosis
3	Discoloration of > two toes
4	Discoloration of one toe
5	Discoloration of > two nails
6	Discoloration of one nail
7	No necrosis

Bibliography:

1. Garcia, S., et al., *Prognostic value of 12-lead electrocardiogram and peak troponin I level after vascular surgery.* J Vasc Surg, 2013. **57**(1): p. 166-72.
2. Tarlov, I.M., *Spinal cord compression studies. III. Time limits for recovery after gradual compression in dogs.* AMA Arch Neurol Psychiatry, 1954. **71**(5): p. 588-97.

1.2 Supplementary Figures

A

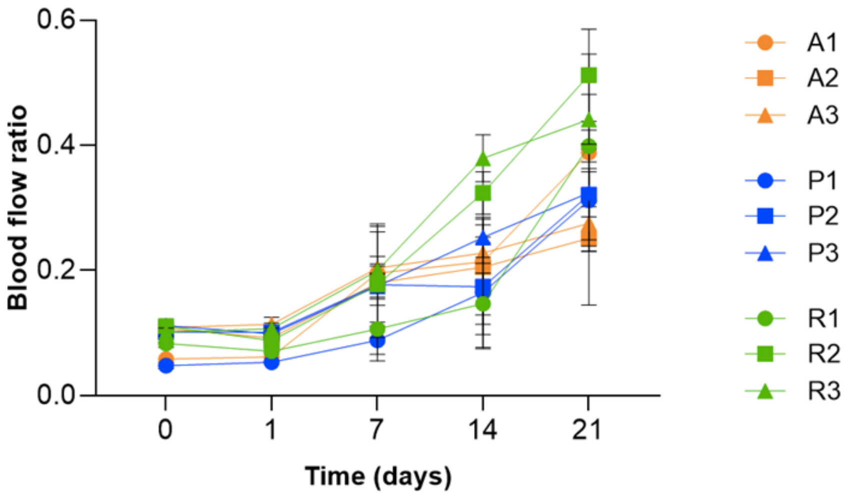

B

Day	0 pre				0 post				1			
	Mean	SEM	%CV	n	Mean	SEM	%CV	n	Mean	SEM	%CV	n
A	1.006	0.015	5.555	14	0.098	0.008	29.704	14	0.101	0.010	35.858	14
P	1.014	0.020	7.704	15	0.097	0.008	31.312	15	0.090	0.007	31.897	15
R	1.025	0.015	6.489	20	0.098	0.005	20.570	20	0.091	0.006	28.769	20

Day	7				14				21			
	Mean	SEM	%CV	n	Mean	SEM	%CV	n	Mean	SEM	%CV	n
A	0.199	0.021	39.070	14	0.221	0.023	38.095	14	0.296	0.037	47.386	14
P	0.158	0.021	52.312	15	0.219	0.031	55.064	15	0.321	0.030	36.081	15
R	0.166	0.019	50.656	20	0.296	0.032	48.916	20	0.446	0.027	27.104	20

Supplementary figure S1. Blood flow evolution per group within time differentiating between donors. A) Individual blow flow ratios per group (A, P, R) per assay (●, ■, ▲) within time are represented. Averaged ratios of left (injured) *vs* right (non-injured) limbs are shown. Measurements were performed on the left and right paws, before surgery (pre) and after surgery (post, day 0), and on days 1, 7, 14 and 21 post-surgery. Groups tested: Adjuvants (A, n:14), Placebos (P, n:15) and REX-001 (R, n:20) treated mice. Three independent assays with three different REX-001 batches were used for this assay. **B)** Statistical data correlated to blood flow changes for the different groups. The averaged values (mean), standard error (SEM), coefficient of variance (CV%) and number of mice (n) per group are shown.

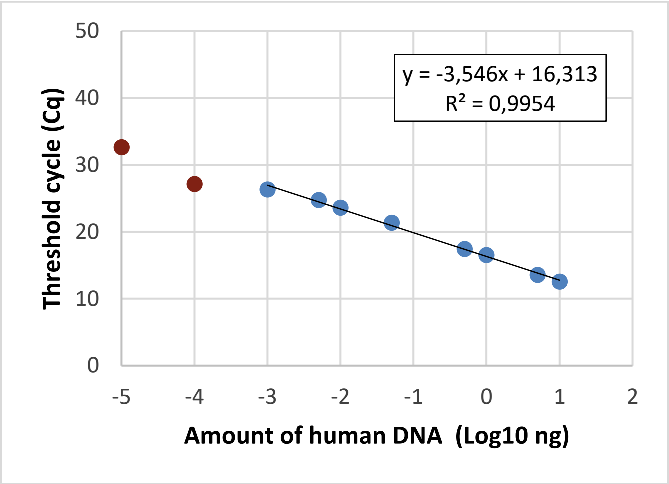

Supplementary Figure S2: Graphical representation of the standard curve obtained to detect human Alu sequences among the mice ischemic tissues. In total, 100 ng of mixed human and mouse genomic DNA were used. Thus, decreasing amounts of human DNA (5,1,0.5, 0.1, 0.05, 0.005, and 0.001 ng), extracted from REX-001 cells, were mixed with increasing amounts of mouse DNA (95,99,99.5, 99.95, 99.990 and 99.999 ng). qPCR was performed using triplicates per simples in one run, and the mean Ct values were plotted. Aditionally, a negative control (NC), with only mouse genomic DNA, and a non-template control (NTC) were used.
